# Fabrication of biodegradable drug-eluting scaffolds by two-photon polymerization

**DOI:** 10.1038/s41598-025-34542-x

**Published:** 2026-01-09

**Authors:** Felix Behlau, Antonio Riveiro Rodriguez, Cemal Esen, Juan Pou, Andreas Ostendorf

**Affiliations:** 1 Applied Laser Technologies, Ruhr University Bochum, Universitätsstraße 150, 44801 Bochum, Germany; 2 Applied Physics Department, University of Vigo, EEI, Lagoas-Marcosende, 36310 Vigo, Spain

**Keywords:** Two-photon polymerization, Drug-eluting, Biodegradable, Scaffold, Medical technology, Biotechnology, Chemistry, Materials science, Nanoscience and technology

## Abstract

Two-photon polymerization (2PP) is a promising fabrication technology for three-dimensional microstructures. The high resolution of 2PP, with the capability to create features down to 100 nm, makes it an attractive tool for medical and biomedical applications. This is enabled by the availability of many biocompatible and biodegradable photoresists for 2PP. Consequently, the fabrication of scaffold structures by 2PP for tissue or cell engineering is a common practice. In this study, a novel approach to scaffold fabrication by 2PP is presented. The incorporation of drugs into a biodegradable photoresist is demonstrated to be a feasible method for passive drug-eluting scaffolds created by 2PP. The drug was mixed into the liquid photoresist prior to processing. Measurement of the drug-release was done by immersing the scaffolds in a water solution for a period of two weeks at 37 °C, with constant shaking in an incubator. The drug release was measured by taking extracts at defined time intervals and measuring the concentration using Inductively Coupled Plasma Mass Spectrometry (ICP-MS). As a result, an initial burst and a subsequent slowing increase of the drug concentration were detected.

## Introduction

Two-Photon Polymerization (2PP) represents the highest resolution additive manufacturing technology^1^, and it has garnered a growing interest in recent years, exhibiting an upward trajectory in annual citations^2^. In 2PP, an ultra-short pulsed laser is focused inside a photosensitive material to induce the quasi-simultaneous absorption of two photons by the photoinitiator^3^. Consequently, the photoinitiator generates free radicals to initiate a free-radical polymerization process, thereby curing the liquid photoresist^4^. Given the necessity of a high photon density to exceed the polymerization threshold due to the nonlinear absorption characteristic, only a very small volume within the laser focus is solidified^5^. By moving the laser inside the photoresist, arbitrary three-dimensional structures with sub-diffraction-limited resolution can be created^6^.

The ability to create arbitrarily shaped three-dimensional structures with a sub micrometer resolution makes 2PP a versatile tool with applications in a multitude of fields, including microoptics^7^, microfluidics^8^, and medical applications^9^. In the medical field in particular, 2PP offers a wide range of applications, including scaffolds for cell culture^10^ or tissue engineering^11^, microneedle arrays^12^, endoscopes^13^, and organs-on-a-chip^14^.

The material used for 2PP can be selected from a wide range of photopolymers with various properties^15^, so that the resulting structures can be tailored specifically to the medical application^16^. A particularly promising category of materials for medical applications are biodegradable polymers. These can be implanted into the body without the need for subsequent removal, as they degrade along with the body’s natural metabolic processes^17^.

Another advantage of 2PP is that the photoresist used is predominantly liquid in its unprocessed form. As a result, additives can be incorporated into the photoresist to achieve additional properties of the cured structure, such as electrical conductivity^18^ or magnetic properties^19^. This characteristic presents a significant opportunity for advancements in the medical field, as it enables the incorporation of drugs into the photoresist, as demonstrated in^20^. When combined with biodegradable materials, this results in a drug release during degradation, as demonstrated for hydrogel-based materials by^19^. However, to date, these approaches have utilized actively released drugs, such as acoustically^21^ and magnetically^22^, or have employed microparticles within the material to carry the drug^23^. In contrast, the incorporation of the drug directly into the biodegradable photoresist could enable passively drug-releasing structures.

This approach could result in a continuous drug release during degradation, which could be used in the future to create biodegradable 3D drug-releasing structures. One potential application could be drug-eluting stents, as this would be a significant improvement over current state-of-the-art drug-releasing stents, which are coated with a drug^24^ and are unable to release any more drug once depleted. Consequently, this could enhance the functionality of additive-manufactured implants in general, which already offer the benefit of specific shapes that are tailored to patients^25^.

This work presents a proof of concept for the feasibility of passively drug-releasing polyester-based biodegradable structures through the fabrication of drug-eluting scaffold structures using 2PP. For comparative purposes, the samples were fabricated out of biodegradable and non-biodegradable photoresists. Different concentrations of a drug were added to both non-cured materials. Subsequently, the structures were immersed in a water solution at 37 °C while constantly shaken for two weeks in an incubator. The drug release was measured by taking extracts at defined time intervals and measuring its drug concentration with Inductively Coupled Plasma Mass Spectrometry (ICP-MS). Furthermore, the samples were investigated using a light microscope and a scanning electron microscope (SEM).

## Experimental setup

The 2PP setup used for the experiments^26^ consisted of a Ti:Sa laser (Tsunami, Spectra-Physics Inc.) providing 100 fs ultra-short laser pulses with a repetition frequency of 82 MHz, and a central wavelength of 780 nm. To adjust the average laser power to the desired level, a $\lambda /2$ - waveplate in combination with a polarizing beamsplitter was employed. The utilized average laser power and all further processing parameters are listed in Table 1. Since the biodegradable and non-degradable photoresists are quite different, it was necessary to use different average laser powers and scan speeds.

In order to fabricate scaffolds with a sufficient size, a microscope objective with a field of view of 1 mm x 1 mm was used (20x NA=0.8 Plan-Apochromat, Carl Zeiss Microscopy Germany GmbH). The fabrication process and sample positioning were performed using a Galvo scanner (hurrySCAN II 14, SCANLAB GmbH) and three linear axes (Wafer Max Z and ANT 130-XY, Aerotech Inc.).

**Table 1:** 2PP processing parameters.

Process parameter	Value	Unit
layer thickness	0.4	$\upmu$m
Hatch distance	0.4	$\upmu$m
Microscope objective field of view	1000 x 1000	$\upmu$m
Average laser power (non-degradable photoresist)	40	mW
Average laser power (degradable photoresist)	70	mW
Laser scan speed (non-degradable photoresist)	50	mm/s
Laser scan speed (degradable photoresist)	300	mm/s

**Fig. 1:**
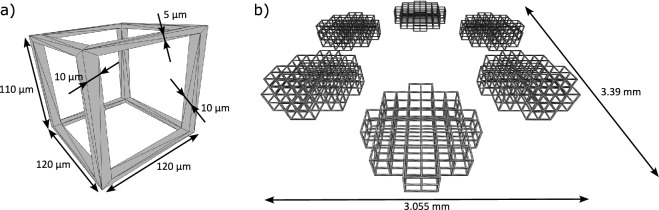
3D-model of scaffold structures. (**a**) unit cell and (**b**) complete sample.

The 3D-scaffold structures were designed using a CAD program (Inventor 2024, Autodesk, Inc.) and exported in the STL file format for further processing in the setup. The dimensions of a single scaffold cell are depicted in Fig 1a, while the complete arrangement of six consecutively fabricated scaffolds is illustrated in Fig. 1b. The unit cell has external dimensions of 120 $\upmu$m x 120 $\upmu$m x 110 $\upmu$m, and the vertical side bars have a thickness of 10 $\upmu$m in both dimensions, while the horizontal bars have a width of 10 $\upmu$m and a thickness of 5 $\upmu$m. Thus, the inner free area is a square with an edge length of 100 $\upmu$m, to allow cells to grow into it for further studies. The horizontal bars have a height of 5 $\upmu$m, which is smaller than the other dimensions of 10 $\upmu$m. This adjustment is necessary to compensate for the elliptical shape of a cured voxel, which results from the wider gaussian intensity distribution in the laser direction compared to the intensity distribution perpendicular to the laser direction. This effect is particularly pronounced for smaller numerical apertures (NA) and smaller magnifications of the utilized microscope objective, such as the one utilized in this study with an NA of 0.8 and a magnification of 20x. Nevertheless, such an objective was necessary, as it also enables a sufficiently large field of view.

## Sample preparation

The biodegradable material utilized in this study was DEGRAD INX (BIO INX, Belgium), which is a long-term degradable material with degradation times of 3 to 5 years upon contact with water. The non-degradable reference material used was SZ2080^27^. The added drug was the disinfectant and antiseptic iodinated povidone (homopolymer from 1-vinyl-2-pyrrolidone, complex with iodine, Sigma-Aldrich). This compound was selected because one of the main challenges in tissue engineering is the creation of scaffolds that promote cell adhesion and proliferation while bacterial colonization is inhibited^28^. In this regard, iodine and compounds are well-known antibacterial agents, used in many antiseptic applications. Moreover, these compounds have not been shown to cause bacterial resistance, which is a serious global health problem that can compromise the success of implants.

A total of six samples were fabricated, utilizing the biodegradable material (DEGRAD INX) and the non-biodegradable material (SZ2080) for comparative purposes. Into both materials, a drug (iodinated povidone) was added in a volume ratio of 1% and 5%, as well as 0% as a reference. The six different samples used in this study are listed in Table 2 as an overview.

**Table 2:** Sample overview.

Number	Photoresist	Added drug (%)
1	SZ2080	0
2	SZ2080	1
3	SZ2080	5
4	DEGRAD INX	0
5	DEGRAD INX	1
6	DEGRAD INX	5

**Table 3:** Volume and weight ratios of added drugs into photoresist.

Sample number	Photoresist	Photoresist volume ($\upmu$l)	Photoresist weight (mg)	Drug volume ($\upmu$l)	Drug weight (mg)	Drug volume ratio (%)	Drug weight ratio (%)
2	SZ2080	200	196.0	2	2.2	1	1.12
3	SZ2080	200	199.4	10	9.9	5	4.96
5	DEGRAD INX	100	66.5	1	0.8	1	1.20
6	DEGRAD INX	80	55.4	4	4.1	5	7.40

Pre-processing was necessary for both photoresists before they could be processed with 2PP. The non-biodegradable SZ2080 required a baking process at  80 °C for 20 minutes. The biodegradable material DEGRAD INX required preheating at 50 °C for 10 minutes. After preheating, the biodegradable photoresist was pipetted onto the glass substrate and baked at 70 °C for 20 minutes. This process was performed to evaporate the solvent and make the photoresist 2PP processable. For all samples, pre-silanized glass cover slips were utilized as a substrate.

Following the processing stage, both photoresists were chemically developed by immersion in a developing solution. For the SZ2080 sample, a solution comprising a 1:1 ratio of 2-propanol and 4-methyl-2-pentanone was utilized. In contrast, acetone was employed as the developing agent for the DEGRAD INX sample.

In order to incorporate the drugs into the photoresist, the drug was mixed into the resists prior to the baking step. The liquid resist was pipetted into an Eppendorf tube with a micropipette to achieve the exact ratios of 1 % and 5 % by volume of the added drug. Subsequently, the precise addition of the drug was executed using the micropipette. To ensure an accurate assessment of the applied volumes, the weight of the added resist and drug was measured in addition to the dispensed volume. This approach accounts for any minor variations in deposited volume due to the photoresists viscosity. The corresponding volumes and measured weights are presented in Table 3. Following the addition of the photoresist and drug into the Eppendorf tube, a magnetic stirrer was employed to mix the contents at a rate of 300 rpm for a duration of 1 hour. Upon completion of the mixing procedure, the photoresist was inspected, which revealed that the photoresist appeared homogeneous as observed both visually and through the 20x microscope objective within the 2PP setup.

To examine drug encapsulation, two additional droplets of the biodegradable photoresist containing 5% of the drug were prepared. Scaffolds were fabricated by 2PP in one droplet, and no processing was done in the other. Then, both samples were developed in 10 ml of aceton. The drug concentration was measured using ICP-OES and the results were compared. The unprocessed droplet exhibited a higher concentration of 1.6 ppm, comparable to the expected amount of drug in the 2PP-processed structures. Therefore, the drug is encapsulated inside the polymerized photoresist. However, since the exact volume of the scaffold structures cannot be determined, the encapsulation efficiency cannot be calculated precisely.

The drug release measurement was performed by immersing the samples in deionized water and incubating them at 37 °C in an orbital shaker under agitation at 120 rpm. Deionized water was utilized as an immersion medium to isolate the effect of hydrolysis from potential ionic interactions that could affect drug solubility. Extracts of the water were taken after 1 h, 4 h, 8 h, 24 h, 72 h, 168 h, and 336 h. These data points were selected within a 14-day interval to observe the early-stage drug release behavior. The drug concentration of these extracts was subsequently measured by ICP-MS (Thermo-Finnigan Neptune).

In addition, the samples were analyzed using a light microscope and a SEM. A before-and-after comparison was conducted using a light microscope because sputtering is necessary for SEM imaging, which would hinder the subsequent drug release of the samples. Consequently, the SEM imaging was performed for all samples after the water immersion experiment. Nevertheless, an additional sample was prepared from the biodegradable photoresist for a before-and-after dissolution test comparison to facilitate an analysis of the degradation.

## Results

**Fig. 2:**
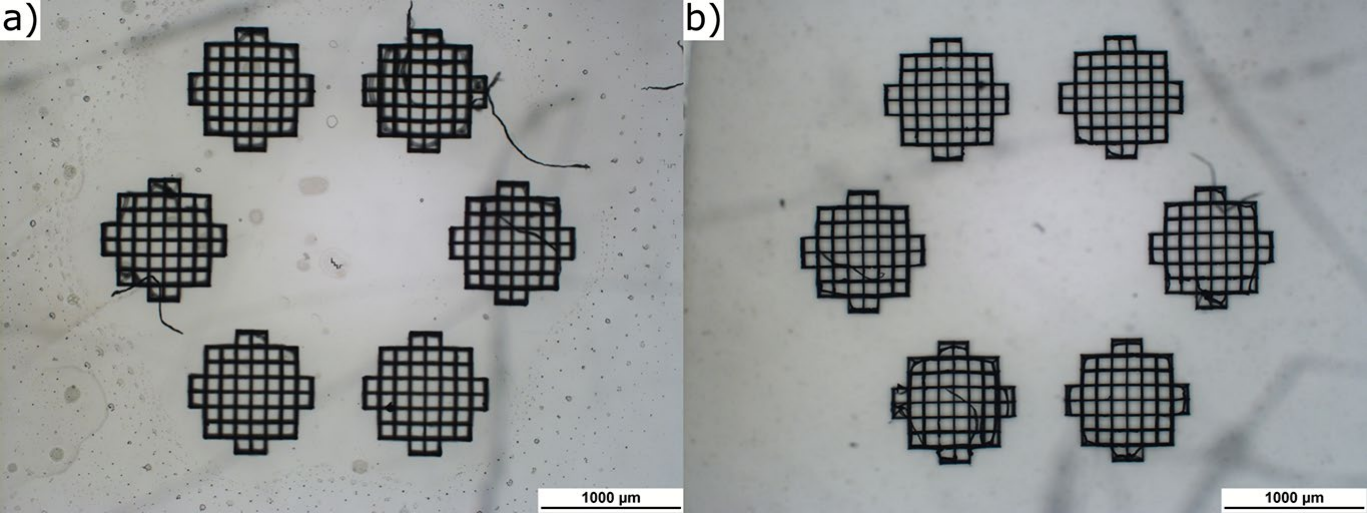
Light microscope images of fabricated scaffolds with biodegradable material with 5 % drug before (**a**) and after (**b**) dissolution test.

The fabrication of one scaffold structure required a 2PP processing time of 42 minutes for the nondegradable material and 37 minutes for the biodegradable material due to the higher scanning speed used with the biodegradable material. In Fig. 2, images captured by a light microscope at a magnification of 2.5x of a sample fabricated with the biodegradable material containing 5 % of the drug are shown. Fig. 2a presents the six scaffolds prior to water immersion, while Fig. 2b illustrates the structures after the two weeks of water immersion. It is evident that the fabrication of the scaffolds with 5 % of the drug was successful. Nonetheless, a noticeable difference between the before and after images is not visible under a light microscope. However, given that the biodegradable material requires 3-5 years to fully degrade when in contact with water, it is expected that the degradation will be minimal and therefore not visible under a light microscope.

**Fig. 3:**
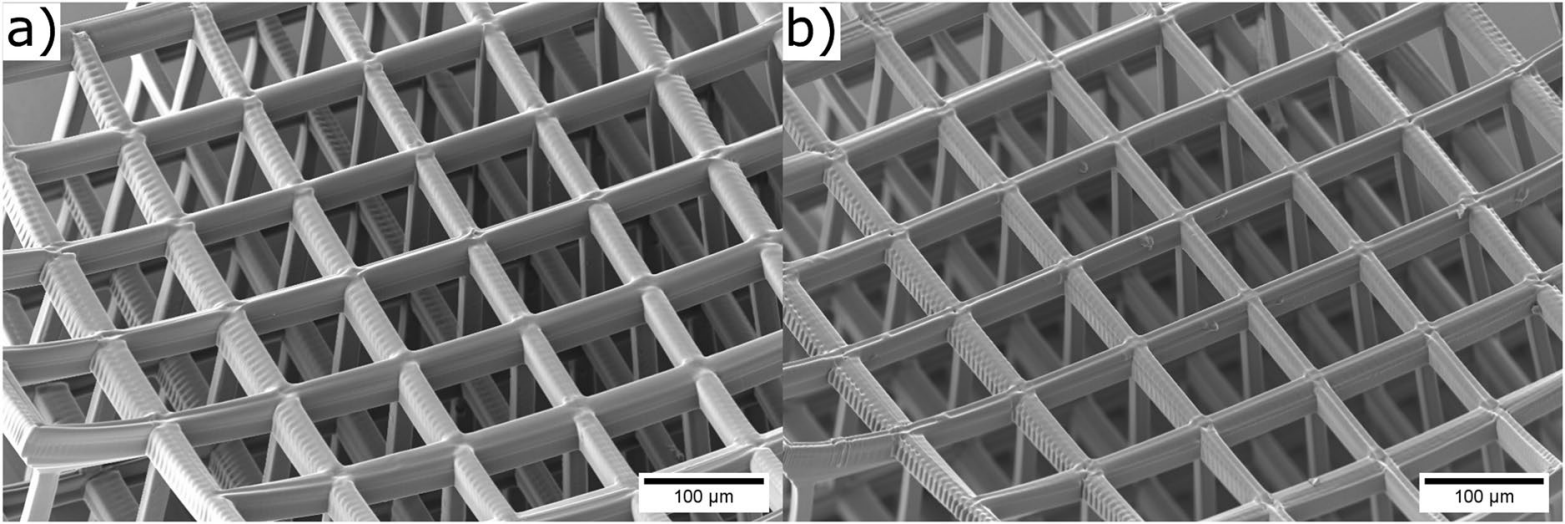
SEM image of a scaffold fabricated with the biodegradable material without an added drug before (**a**) and after (**b**) the dissolution test.

In Fig. 3, SEM images of the biodegradable material without added drug are shown, both before and after the dissolution test. As illustrated in Fig. 3a, which was taken prior to the immersion in water, the bars appear slightly thicker than in Fig. 3b, which was taken afterwards. However, no evident indications of degradation are visible, which is consistent with the anticipated 3-5 year degradation time of the photoresist.

**Fig. 4:**
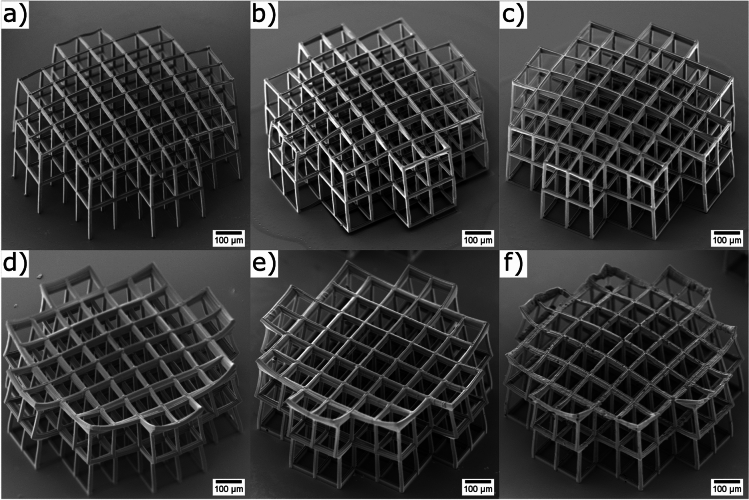
SEM images of scaffolds. Non-degradable material with 0 % (**a**) 1 % (**b**) and 5 % (**c**) of the drug added and biodegradable material with 0 % (**d**) 1 % (**e**) and 5 % (**f**) of the drug added.

Figure 4 shows the SEM images of the scaffolds after water immersion. Here, the scaffolds fabricated with the non-degradable material are displayed in Fig. 4a–c, where 0 % (a), 1 % (b), and 5 % (c) of the drug are added. As a comparison, the scaffolds fabricated with the biodegradable material are shown in Fig. 4d–f, where (d) contains 0 %, (e) contains 1 %, and (f) contains 5 % of the drug. It is evident from these figures that the two materials exhibit divergent field curvatures. The surface of the non-degradable scaffold is convex, while the degradable material is concave. This is noteworthy, as both materials were fabricated under identical conditions and with the same laser movement paths. Given that both materials were fabricated in a configuration in which the laser irradiates through the glass inside the photoresist, they were printed from the top layer of the structure to the bottom layer at the glass surface. This printing method was employed to prevent the laser from passing through already polymerized material. Consequently, the top layers were printed first, as the material has a high viscosity after the baking step, allowing unpolymerized material to support cured material. However, slight deformations resulting from material contraction or expansion remain a possibility, with the potential to induce these slight changes in shape.

Furthermore, it is evident that the 2PP processability of the biodegradable material is reduced with increased drug concentration. This phenomenon is particularly noticeable in Fig. 4d–f, where the images with a higher percentage of drug have more holes and processing defects. Additionally, the upper left corner of Fig. 4f appears to be partially non-polymerized. One potential reason for this is that the increased drug concentration lowers the photoinitiator concentration. In addition, the presence of small drug particles within the photoresist can lead to an increase in light scattering, thereby reducing the intensity within the laser focal volume. However, it is important to note that both effects should also occur for the non-degradable material, but they are not visible in the Fig. 4c. Furthermore, it is noteworthy that these effects could be compensated by increasing the laser power.

**Fig. 5:**
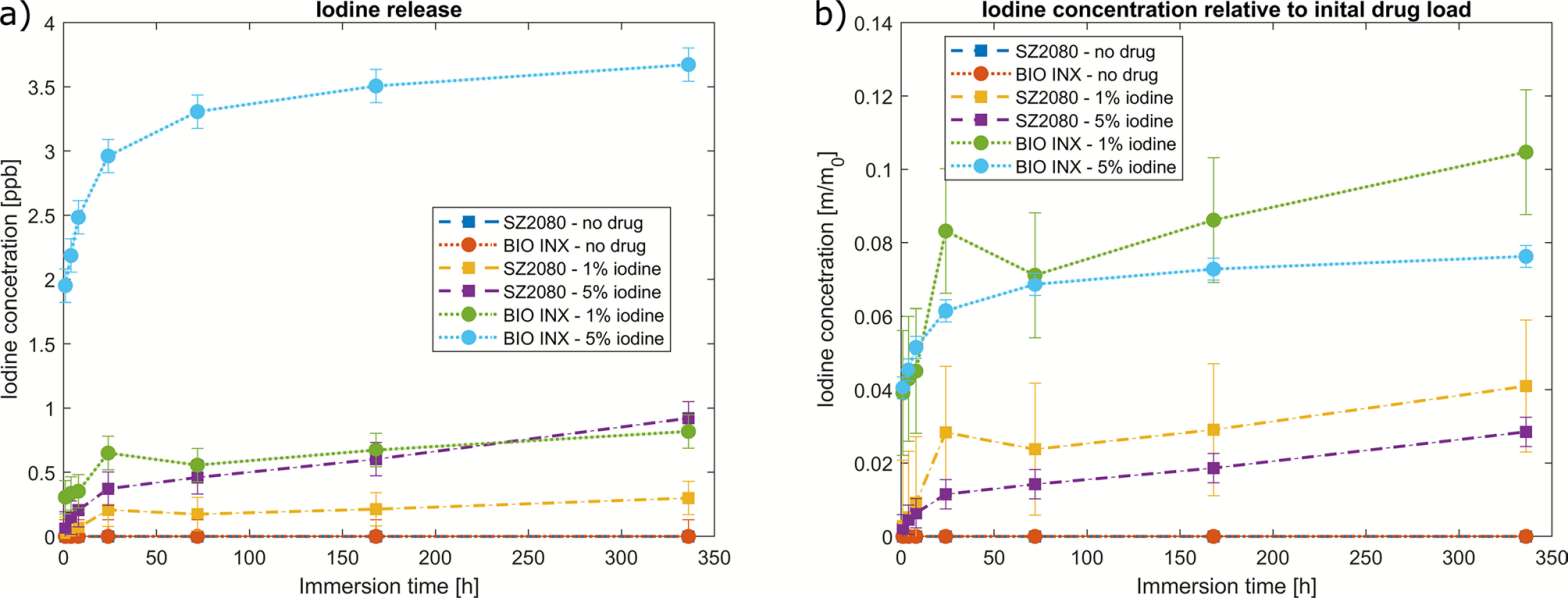
Concentration of released drug over time.

The concentration of the drug iodinated povidone released into the water was measured by ICP-MS and is shown in Fig. 5a. The error bars represent the standard deviation of the ICP-MS measuring device. It is evident that the biodegradable photoresist DEGRAD INX mixed with 5 % iodinated povidone exhibited the most significant drug release into the water. The maximum concentration that was measured was 3.5 ppb (parts per billion). In contrast, the sample with only 1 % drug added reached a maximum concentration of about 0.75 ppb. As expected, the sample with no drug added showed no release. It is notable that the non-degradable material SZ2080 also released a small amount of drug, 0.25 ppb for 1 % and 1 ppb for 5 %. This observation indicates that the release of a minimal amount of drug into the water environment is possible without material degradation. This phenomenon may be attributed to diffusion processes or the dissolution of small amounts of drug solidified at the outer edges of the scaffold structures during the 2PP process. Moreover, the incorporation of the drug into the material could potentially lead to a less effective cross-linking of the material, resulting in additional release of the drug into the water.

As illustrated in Fig. 5b, the quantity of released drug is expressed as a percentage of the calculated initial drug load. It can be observed that the biodegradable material has released a higher percentage of the initial drug compared to the non-degradable material. It is noteworthy that for both materials, the sample with 1% of the drug exhibited a higher ratio of the initial drug released than the 5% sample. Due to the observation that the 5% biodegradable samples exhibited a substantially higher occurrence of structural defects in comparison to the 1% sample, it can be proposed that the predominant mechanism of drug release in this instance is not through diffusion or insufficient cross-linking but rather biodegradation.

The observed release patterns, characterized by an initial burst followed by a slowing increase, are typical for drug release when the drug is dispersed inside a material^29^. It is important to note that the constant release of iodine over a prolonged period guarantees a permanent antibacterial effect.

## Conclusion

In this work passively drug-eluting biodegradable structures fabricated by 2PP, which released the drug directly without a secondary phase, were successfully demonstrated. The drug iodinated povidone was successfully mixed into the liquid biodegradable photoresist DEGRAD INX and subsequently processed to fabricate scaffold structures. The drug-release was detected by immersing the scaffold structures in a water solution and shaking them at 37 °C for two weeks. During this period, multiple extracts of the water were taken, and the drug concentration was measured by ICP-MS. These measurements showed an absolute drug concentration of 3.5 ppb for the structures fabricated out of the biodegradable photoresist with 5% iodinated povidone.

Additionally, the degradable sample with only 1% added drug exhibited a higher relative drug release compared to the 5% sample. Moreover, the biodegradable samples exhibited a significantly higher rate of drug release in comparison to the non-degradable material. Therefore, it can be hypothesized that the primary contribution to the release was attributable to degradation. However, diffusion processes and the presence of non-fully cured material could have also contributed to the observed drug release. Furthermore, a constant increase in drug concentration was observed over the course of the two-week experiment.

Moreover, it was observed that a reduced drug dosage in the unprocessed photoresist also results in a lower drug concentration in the water. This observation allows the amount of drug released to be adjusted by modifying the initial dosage before processing. Nevertheless, given that an elevated drug load also results in a worsened polymerization reaction, further studies are necessary to determine the maximum amount of drug that can be added before the material becomes incapable of undergoing the polymerization process.

In addition to adjusting the dosage of the drug in the untreated photoresist, it is possible to increase the total released drug concentration by selecting a photoresist that degrades more rapidly or using 3D structures with a larger surface area to facilitate stronger drug release.

The approach demonstrated in this work shows passive drug release of biodegradable micro-scaffolds with potential applications in tissue engineering and regenerative medicine.

## Data Availability

The data that supports the findings of this study is available from the corresponding author, FB, upon reasonable request.
